# Comparing Effectiveness of Combination of Collagen Peptide Type-1, Low Molecular Weight Chondroitin Sulphate, Sodium Hyaluronate, and Vitamin-C Versus Oral Diclofenac Sodium in Achilles Tendinopathy: A Prospective Randomized Control Trial

**DOI:** 10.7759/cureus.19737

**Published:** 2021-11-19

**Authors:** Arun Choudhary, Samantak Sahu, Abhimanyu Vasudeva, Nishat Ahmed Sheikh, Srikumar Venkataraman, Gita Handa, Sanjay Wadhwa, Upinderpal Singh, Shivanand Gamanagati, S. L Yadav

**Affiliations:** 1 Physical Medicine and Rehabilitation, All India Institute of Medical Sciences, Jodhpur, Jodhpur, IND; 2 Physical Medicine and Rehabilitation, All India Institute of Medical Sciences, Gorakhpur, Gorakhpur, IND; 3 Forensic Medicine and Toxicology, All India Institute of Medical Sciences Gorakhpur, Gorakhpur, IND; 4 Physical Medicine and Rehabilitation, All India Institute of Medical Sciences, New Delhi, New Delhi, IND; 5 Physical Medicine and Rehabilitation, Mahatma Gandhi Hospital, Mahatma Gandhi University of Medical Sciences and Technology, Jaipur, IND; 6 Radiology, All India Institute of Medical Sciences, New Delhi, New Delhi, IND; 7 Physical Medicine and Rehabilitation, All India Institute of Medical Sciences New Delhi, New Delhi, IND

**Keywords:** ultrasound, vas, vitamin - c, sodium hyaluronate, chondroitin sulphate, collagen peptide type 1, achilles tendinopathy

## Abstract

Background

Achilles tendinopathy, a common cause of heel pain, is primarily considered mechanical in origin, but its pathogenesis and treatment lack consensus. Molecules such as collagen peptide type-1, low molecular weight chondroitin sulphate, sodium hyaluronate and vitamin C have been shown to act as building blocks of tendon structure, and oral supplementation of these have promising results in Achilles tendinopathy.

Methods

This study was a prospective randomized control trial to compare the effectiveness of oral diclofenac sodium versus a nutraceutical combination of collagen peptide type-1, chondroitin sulphate, sodium hyaluronate, and vitamin C in the treatment of Achilles tendinopathy on pain and ultrasonographic structures. A total of 40 patients satisfying inclusion and exclusion criteria were randomly allocated into two groups and were given the nutraceutical combination in group A and diclofenac sodium in group B. The patient evaluation was done at baseline, six-week, and 12-week intervals in terms of VAS (Visual Analogue Scale) and tendo-Achilles thickness by ultrasound.

Results

Both nutraceutical combination and diclofenac reduced pain in persons with Achilles tendinopathy. The nutraceutical combination had a statistically significant better outcome in reducing pain at the end of 12 weeks. On ultrasound, both the interventions reduced Achilles tendon anteroposterior and mediolateral thickness by the end of 12 weeks. Although there was no absolute significant intergroup difference, the percentage change was more in the nutraceutical group in the case of anteroposterior thickness.

Conclusion

Combining collagen peptide type-1, low molecular weight chondroitin sulphate, sodium hyaluronate, and vitamin C is more effective than oral diclofenac in controlling pain in Achilles tendinopathy.

## Introduction

The common degenerative diseases that occur in tendons are known as tendinopathies. They are characterized by activity-related pain, focal tenderness, and decreased strength and movement in the affected tendon and can virtually occur in any tendon [1]. Although the pathological process of tendinopathies is multifactorial, even now, a complete understanding of these processes is not known. Despite several treatments and modalities suggested, there is a lack of pathological correlation and inconsistency in clinical outcomes [2].

Treatment strategies have primarily focused on the control of inflammation. The specific treatment modalities include rest, nonsteroidal anti-inflammatory drugs (NSAIDs), and peri-tendon corticosteroid injections. Exercises such as eccentric training have also been shown to produce results [2,3].

Achilles tendinopathy is frequently encountered in clinical practice. It is important to note that tendinopathy may involve insertional and non-insertional areas of the tendon [4]. The etiopathology of Achilles tendinopathy can be explained by a web of causation, including both intrinsic and extrinsic factors. Ineffective healing and connective tissue degeneration are commonplace. The inadequate repair shows three overlapping clinical stages: reactive tendinopathy, tendon disrepair, and degenerative tendinopathy [5]. In the past several years, oral nutritional supplements have been used to streamline the turnover of tendon connective tissue to prevent ineffective healing. Oral supplements of glucosamine and chondroitin sulphate [6], vitamin C [7], hydrolyzed type 1 collagen [8], and sodium hyaluronate [9, 10] increase the concentration of these building blocks of tendon and might facilitate to conserve, or even repair, the degenerated tendon connective tissues. This study hypothesized that the nutraceutical combination (collagen peptide type-1, low molecular chondroitin sulphate, sodium hyaluronate, and vitamin C) would fare better than diclofenac sodium concerning pain and ultrasound findings.

## Materials and methods

This prospective randomized controlled study investigated the effectiveness of this nutraceutical combination (clinical and ultrasound) compared to diclofenac sodium in Achilles tendinopathy. The study was conducted at the Department of Physical Medicine and Rehabilitation (PMR) in a tertiary care hospital. The study was started after approval from the Institute Ethics Committee for Postgraduate Research, All India Institute of Medical Sciences, New Delhitill the required sample size was attained (approval number IESC/T-147/01.04.2015). The period of study was July 2015 to November 2016. The study was registered in the Clinical Trials Registry of India (CTRI/2020.06/026230).

All subjects with posterior heel pain attending PMR outpatient service were clinically examined for Achilles tendinopathy and confirmed by USG during the above-mentioned period, fulfilling the inclusion and the exclusion criteria were allocated randomly either in the study or control group. Randomization was done by computer software (Research Randomizer, Social Psychology Network). The sample size arrived at 20 patients in each group based on an unpublished pilot study conducted in the same department. The sample size was calculated based on the mean difference in VAS between a nutraceutical and diclofenac treated group at 12 weeks of administration of study medications. The parameters considered were: expected mean difference of 10 mm, the pooled value of standard deviation - 10 mm, power (β) - 80% and level of significance (α)- 0.05. The calculated sample size from the online tool was 16. A dropout rate of 20% was assumed to calculate the sample size. Informed written consent in Hindi/English and explained in the patient's own language was taken from all the patients willing to participate in this study. This study was carried out according to good clinical practice and the Declaration of Helsinki. [11]

Patients aged 18 years or over of either gender, physical examination revealing maximum tenderness at the attachment of Achilles tendon on the posterior aspect of the calcaneus or anywhere along its entire length, patients diagnosed clinically and confirmed by USG, with unilateral or bilateral Achilles' tendinopathy of any duration were included in the study.

Patients more than 75 years of age, ultrasound suggestive of complete rupture of Achilles tendon or retrocalcaneal bursitis, history of any local trauma or infection or autoimmune enthesitis, having received local steroid injection or similar drug combination of collagen peptide type-1, low molecular weight chondroitin sulphate, sodium hyaluronate and vitamin C within three months were excluded. A diagnosis of vascular insufficiency or neuropathy related to posterior heel pain (radiculopathy, tarsal tunnel syndrome) and other causes of posterior heel pain were also excluded. Hip or ankle instability, history of intra-articular joint injection in the ankle joint within the past three months of the study were excluded. Other reasons for exclusion were comorbid conditions such as uncontrolled diabetes mellitus, chronic renal failure, connective tissue disorders, pregnancy, and lactation.

The diagnosis of Achilles tendinopathy was made based on history, clinical examination, and ultrasound examination.

The study group received a nutraceuticalcombination comprising of collagen peptide type-1 (40 mg), chondroitin sulphate (200 mg), sodium hyaluronate (30 mg) and vitamin C (35 mg) in a dose of one tablet thrice a day for 12 weeks. In the control group, diclofenac sodium 50 mg in the dose of one tablet twice in a day, if the weight of the patient ≤ 60 kg, or thrice in a day, if the weight of patient > 60 kg was prescribed regularly for 14 days initially, and then on as and when required basis and exact numbers of tablets were recorded in a diary by the patient as tablet count and any other associated side effects were noted meticulously.

**Figure 1:**
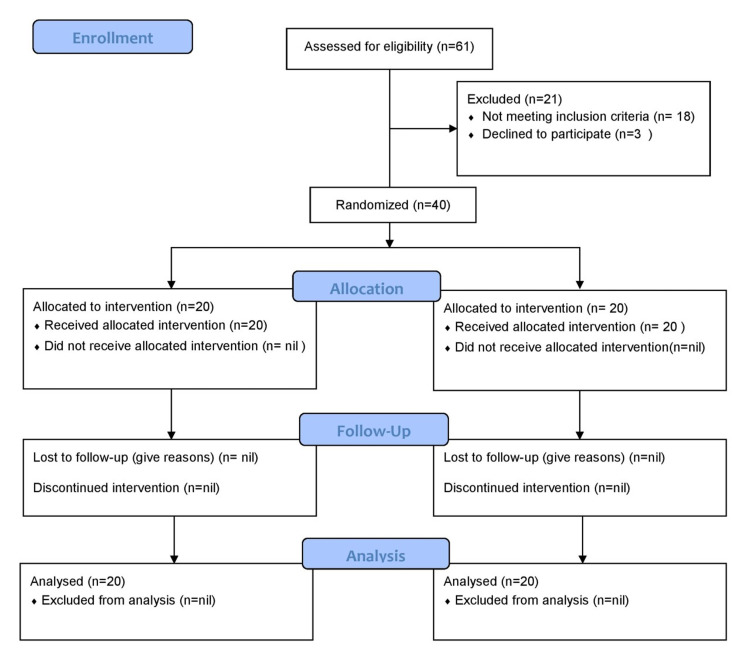
CONSORT 2010 flow diagram Source: http://www.consort-statement.org/consort-statement/flow-diagram CONSORT – CONsolidated Standards Of Reporting Trials

Both groups were evaluated based on pain intensity assessment by visual analogue scale (VAS) and USG assessment at baseline, six weeks, and 12 weeks. Both groups were given half an inch of heel raise for offloading and resting the Achilles tendon. Both groups were given lifestyle and occupational modifications advice and home-based exercise programs, including stretching exercises and eccentric strengthening exercises, for 12 weeks. If the symptoms were exacerbated, patients were advised to reduce the number of repetitions in an eccentric exercise program or rest and suggested cold application. Patients included in the study for more than six weeks of duration not responding to any of the two treatments (pain affecting activities of daily living) were considered a failure of treatment and were taken as endpoint and considered for alternate treatment. Patients were contacted weekly via telephone by a resident physiatrist to encourage them to continue the exercise program. In case of any difficulty, exercise techniques were checked by video call wherever possible and appropriate instructions were provided (Figure 1).

The eccentric exercise program had a frequency of 15 repetitions thrice daily (total of 45 repetitions). Demonstration of the exercise was given to each patient for better compliance with the exercise program. The intensity of exercise was gradually increased by augmenting the number of repetitions for the first 1 to 2 weeks to achieve the desired frequency of 15 repetitions thrice in a day as comfort allowed and was maintained up to the 12th week.

Outcome measures

Pain Intensity

Pain was assessed with a numerical visual analogue scale (VAS) of 0 to 100, in which 0 represents no pain and 100 represents the worst pain in each subject. The VAS was self-completed by the patient. The patient was asked to place a line perpendicular to the VAS line to represent their pain intensity. Using a ruler, the score was determined by measuring the distance (mm) on the 100 mm line between the "no pain" anchor and the patient's mark, providing a range of scores from 0-100mm.

USG Evaluation at Baseline, 6th and 12th Week

**Figure 2:**
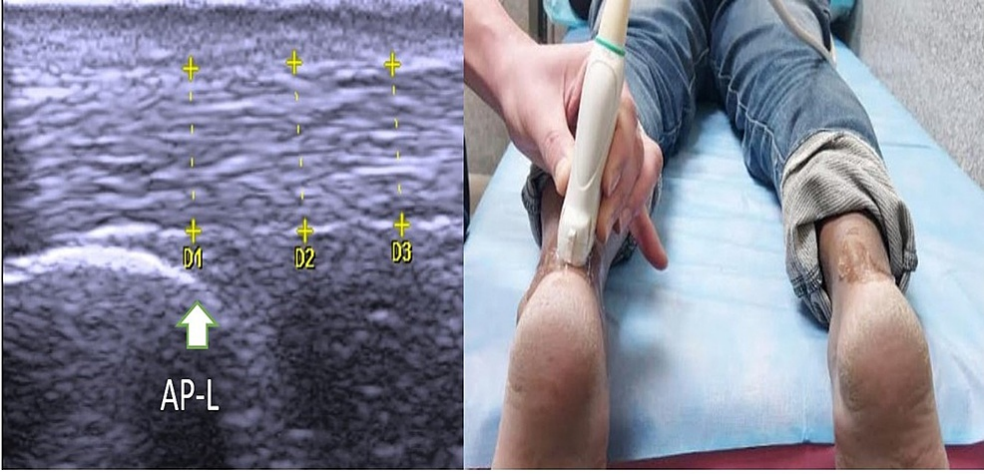
Anteroposterior thickness of Achilles tendon with longitudinal probe position (AP-L)

**Figure 3:**
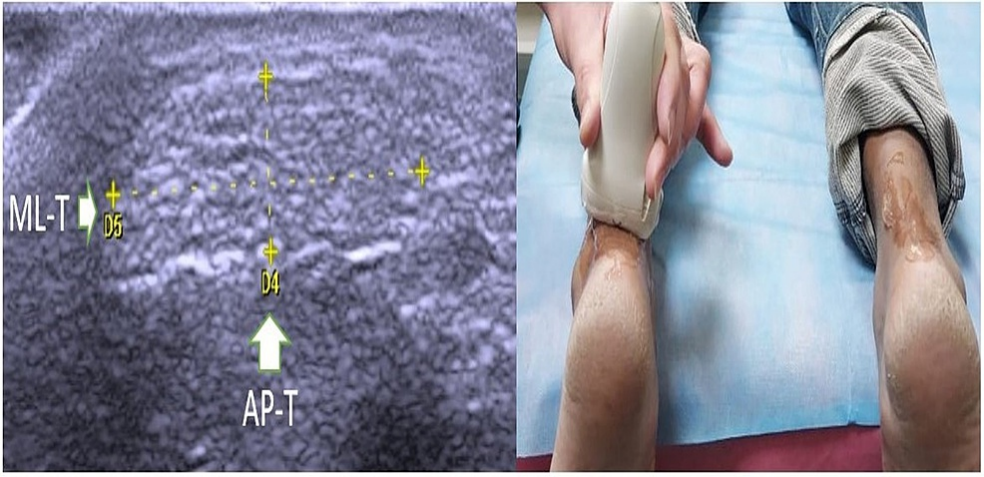
Anteroposterior thickness of Achilles tendon with transverse probe position (AP-L) and mediolateral Achilles tendon thickness (ML-T)

Ultrasonography was performed with the ultrasound machine's 6-13 MHz linear-array transducer (My Lab One, Esaote, Genoa, Italy). Ultrasound examinations were performed on all patients by a resident physiatrist under the guidance of a consultant radiologist at every visit. The anteroposterior diameter of the Achilles tendon was measured on a longitudinal view (AP-L) (Figure 2) and transverse view (AP-T) (Figure 3) with the patient lying prone and both their feet out from the margin of the bed while keeping the ankle in the neutral position. Mediolateral diameter (ML-T) (Figure 3) was taken in a transverse view in the same position. All measurements were taken at the part just proximal to the calcaneum.

The patients were followed up after a period of 6 and 12 weeks. The study was investigator-blinded. A physiatrist with 35 years of experience did patient recruitment. A statistician carried out statistical planning and randomization. Randomization was concealed with prefilled sealed envelopes. A resident physiatrist carried out clinical and ultrasound assessments under the guidance of a senior radiologist with 30 years of experience. Data was compiled and tabulated by a separate resident physiatrist who was unaware of the allocation of groups. Another statistician analyzed the data. Patients could not be blinded due to the different nature of the dosing schedule of the two medications. We delivered tablets to the patients in unlabeled airtight boxes. However, the appearance of tablets was different.

Statistical analysis

Participants were assessed using VAS and USG assessment, and data were summarized and analyzed using SPSS 21 (Released 2012. IBM SPSS Statistics for Windows, Version 21.0. Armonk, NY: IBM Corp.). Qualitative data were expressed in numbers and percentages. Quantitative data were expressed as mean± standard deviation (SD) or median and interquartile range (IQR) as appropriate. Data were tested for normality using the Kolmogorov-Smirnov test. Friedman test was used for within-group analysis and Mann-Whitney U test for between-group analysis when data were not normally distributed. A post hoc analysis was done using the Nemenyi test. A value of p less than 0.05 was considered to represent the statistical significance of the study.

## Results

Baseline parameters were comparable in both groups. Mean BMI in both groups was in the overweight range for the South Asian population [12] (Table 1).

**Table 1:** Baseline parameters

Parameters	Group		p-value
	Nutraceutical (n = 20)	Diclofenac (n = 20)	
Age (Years)	42.75 ± 12.76	42.45 ± 10.52	0.936
Gender			1.000
Male	11 (55.0%)	11 (55.0%)	
Female	9 (45.0%)	9 (45.0%)	
Weight (Kg)	67.25 ± 7.81	63.25 ± 3.89	0.211
Height (cm)	165.35 ± 8.11	160.85 ± 6.82	0.065
BMI (Kg/m2)	24.59 ± 2.20	24.50 ± 1.72	0.925
Duration	14.05 ± 9.42	12.50 ± 7.37	0.724
Side			0.337
Left	7 (35.0%)	10 (50.0%)	
Right	13 (65.0%)	10 (50.0%)	

Within the group, there was a statistically significant improvement. The two groups did not differ in terms of VAS except at 12 weeks. At 12 weeks, reduction in pain according to VAS was significantly more in the nutraceutical group (Table 2).

**Table 2:** Comparison of the two groups in terms of change in VAS over time (n = 40) VAS: visual analogue scale; IQR: interquartile range

VAS	Group		P-value for comparison of the two groups at each of the timepoints (Mann-Whitney U Test)
	Nutraceutical	Diclofenac	
	Median (IQR)	Median (IQR)	
Baseline	80.00 (10.00)	80.00 (10.00)	0.715
6 Weeks	70.00 (10.00)	70.00 (20.00)	0.225
12 Weeks	40.00 (12.50)	60.00 (40.00)	0.031
P-value for change in VAS over time within each group (Friedman Test)	<0.001	<0.001	

There was a significant improvement within the group, but the two groups did not differ in USG (AP-L) (Table 3) at any time point. To explore further, we analyzed the mean of absolute and percentage changes of USG (AP-L) from the baseline to 4 weeks and 12 weeks (Table 4). At baseline to 12 weeks, USG (AP-L) reduction was significantly more in the nutraceutical group.

**Table 3:** Comparison of the two groups in terms of change in USG (AP-L) (in mm) over time (n = 40) USG: ultrasonography; IQR: interquartile range; AP-L: anteroposterior - longitudinal view

USG (AP-L)	Group		P-value for comparison of the two groups at each of the time points (Mann-Whitney U Test)
	Nutraceutical	Diclofenac	
	Median (IQR)	Median (IQR)	
Baseline	6.24 (1.35)	6.38 (1.00)	0.705
6 Weeks	6.00 (1.05)	6.07 (1.20)	0.903
12 Weeks	5.52 (0.97)	5.73 (1.27)	0.655
P-value for change in USG (AP-L) over time within each group (Friedman Test)	<0.001	<0.001	

**Table 4:** Mean change in USG (AP-L) (in mm) from the baseline timepoint to the various follow-up timepoints USG: ultrasonography; AP-L: anteroposterior - longitudinal view

Timepoint Comparison	Change in USG (AP-L) from baseline to follow-up time points						Comparison of the two groups in Terms of Difference of USG (AP-L) from baseline to follow-up time points	
	Group: Nutraceutical			Group: Diclofenac				
	Mean (SD) of absolute change	Mean (SD) of % change	P-value of change within group	Mean (SD) of absolute change	Mean (SD) of % change	P-value of change within group	P-value of absolute change	P-value of % change
4 Weeks - Baseline	-0.63 (0.80)	-8.1% (7.0)	0.010	-0.28 (0.20)	-4.4% (2.9)	<0.001	0.099	0.062
12 Weeks - Baseline	-0.96 (0.99)	-12.7% (7.9)	<0.001	-0.50 (0.32)	-8.0% (5.1)	<0.001	0.029	0.017

In intragroup analysis, there was a significant improvement in both groups. However, the two groups did not differ in USG (AP-T) (Table 5) at any time points. We analyzed the mean of absolute and percentage changes of USG (AP-T) from baseline to 4 weeks and 12 weeks (Table 6).

**Table 5:** Comparison of the two groups in terms of change in USG (AP-T) (in mm) over time (n = 40) USG: ultrasonography; IQR: interquartile range; AP-T: anteroposterior - transverse view

USG (AP-T)	Group		P-value for comparison of the two groups at each of the time points (Mann-Whitney U Test)
	Nutraceutical	Diclofenac	
	Median (IQR)	Median (IQR)	
Baseline	6.06 (1.52)	5.96 (0.91)	0.616
4 Weeks	5.87 (1.20)	5.82 (0.87)	0.946
12 Weeks	5.44 (1.04)	5.72 (1.00)	0.499
P-value for change in USG (AP-T) over time within each group (Friedman Test)	<0.001	<0.001	

**Table 6:** Mean change in USG (AP-T) from the baseline timepoint to the various follow-up timepoints USG: ultrasonography; AP-T: anteroposterior - transverse view

Timepoint Comparison	Change in USG (AP-T) from baseline to follow-up timepoints						Comparison of the two groups in terms of difference of USG (AP-T) from baseline to follow-up time points	
	Group: Nutraceutical			Group: Diclofenac				
	Mean (SD) of absolute change	Mean (SD) of % change	P-value of change within group	Mean (SD) of absolute change	Mean (SD) of % change	P-value of change within group	P-value of absolute change	P-value of % change
4 Weeks - Baseline	-0.50 (0.43)	-7.0% (4.8)	0.003	-0.25 (0.29)	-3.9% (4.2)	0.038	0.048	0.062
12 Weeks - Baseline	-0.83 (0.52)	-12.0% (5.7)	<0.001	-0.47 (0.49)	-7.6% (8.0)	<0.001	0.025	0.028

At baseline to 12 weeks, USG (AP-T) reduction was significantly more in the nutraceutical group. The percentage change of AP-T and AP-L was significantly better in the nutraceutical group at 12 weeks. However, in the case of the mediolateral thickness (ML-T), there was no difference in the two groups when the absolute (Table 7) or percentage change were compared (Table 8).

**Table 7:** Comparison of the two groups in terms of change in USG (ML-T) over time (n = 40) USG: ultrasonography; IQR: interquartile range; ML-T: mediolateral thickness

USG (ML-T)	Group		P-value for comparison of the two groups at each of the timepoints (Wilcoxon-Mann-Whitney U Test)
	Nutraceutical	Diclofenac	
	Median (IQR)	Median (IQR)	
Baseline	1.82 (0.28)	1.67 (0.15)	0.336
4 Weeks	1.65 (0.25)	1.65 (0.20)	0.616
12 Weeks	1.66 (0.18)	1.60 (0.18)	0.370
pvP Value for change in USG (MI-T) over time within each group (Friedman Test)	<0.001	<0.001	

**Table 8:** Mean change in USG (MI-T) from the baseline timepoint to the various follow-up timepoints

Timepoint comparison	Change in USG (MI-T) from baseline to follow-up timepoints						Comparison of the two groups in terms of difference of USG (MI-T) from baseline to follow-up timepoints	
	Group: Nutraceutical			Group: Diclofenac				
	Mean (SD) of absolute change	Mean (SD) of % change	P-value of change within group	Mean (SD) of absolute change	Mean (SD) of % change	P-value of change within group	P-value of absolute change	P-value of % change
4 Weeks - Baseline	-0.10 (0.12)	-4.9% (6.1)	0.016	-0.06 (0.09)	-3.3% (4.8)	0.118	0.498	0.561
12 Weeks - Baseline	-0.15 (0.11)	-7.8% (5.5)	<0.001	-0.13 (0.10)	-7.3% (5.8)	<0.001	0.607	0.665

## Discussion

The purpose of the study was to compare the effectiveness of a nutraceutical combination (collagen peptide type-1, low molecular weight chondroitin sulphate, sodium hyaluronate, and vitamin-c) versus diclofenac sodium in the treatment of Achilles tendinopathy.

Both nutraceutical combination and diclofenac reduced pain in persons with Achilles tendinopathy. The nutraceutical combination had a better result at the end of 12 weeks, indicating its better effectiveness over a more extended period. On ultrasound evaluation, both the interventions reduced Achilles tendon anteroposterior (AP-T, AP-L) and mediolateral thickness (ML-T) by the end of 12 weeks. In intergroup analysis, there was no significant difference between the groups. To evaluate further, we performed post hoc analysis to compare the percentage change in USG parameters (AP-T, AP-L, and ML-T) from baseline to 6 weeks and 12 weeks. Percentage change of AP-T and AP-L was significantly better in the nutraceutical group at 12 weeks. However, in the case of the mediolateral thickness (ML-T), there was no difference in the two groups when the percentage change was compared.

There are multiple treatment strategies for Achilles tendinopathy. Most have limited evidence and suboptimal effectiveness. Oral supplements of glucosamine and chondroitin sulphate [6], vitamin C [7], hydrolyzed type 1 collagen [8] have been used to achieve a cost-effective and non-invasive solution with promising results. A clinical study comparing the effects of injectable glucosamine and indomethacin in the management of Achilles peri-tendinitis showed that glucosamine had a better overall therapeutic effect on two-thirds of the patients, especially in those patients who endured persistent pain [13]. One recent systematic review evaluating the commercially available dietary supplement composed of tendon-specific mucopolysaccharides, type 1 collagen, and vitamin C in the management of different tendinopathy showed improvements on structural improvement in degenerated tendons. They declared the result encouraging while emphasizing the need for more clinical studies [14]. A recent trial also demonstrated that oral collagen peptide supplementation could enhance the effect of structured strengthening programs to reduce pain and return-to-running in persons with chronic Achilles tendinopathy compared to exercise alone. However, it was a small pilot study, and they reiterated the need for full-fledged randomized controlled studies [15].

Our study evaluated the effectiveness of two treatments in terms of relief in pain and the thickness of the Achilles tendon as measured by ultrasound. However, ultrasonographic changes did not reach statistical significance, and the power of the study was not calculated for this. The tendon's thickness was taken as an outcome measure as it is a specific objective parameter. While performing ultrasound examinations, we noticed several other parameters like hypoechogenic zones, loss of fibrillar patterns, irregularity of margins in many of the patients. These changed along with the history of the disease as patients were followed up. Objective scales are lacking to compare these ultrasonographic patterns over a while. Future research may be carried out to develop such accurate scales that can better represent healing responses in Achilles tendinopathy.

The strength of our study was that we combined subjective outcome measures like pain with objective outcome measures like ultrasonography. Moreover, perhaps owing to regular telephonic monitoring of the exercise program, we did not have any loss to follow up and discontinuation of therapy, minimizing the risk of selection bias.

Our study was of 12 weeks duration, so long-term results and recurrence rate could not be assessed. We selected a fixed-dose combination as its components have been shown to target different elements of tendon structure. Participants could not be blinded due to the different dosing schedules of two interventions, which may be a potential source of bias. However, this approach could not reflect the effectiveness of any one of the components of the nutraceutical combination. Future long-term studies may elucidate these factors, and keeping ultrasonographic features as the primary objective may reveal any meaningful changes in Achilles tendon thickness before and after the treatment.

## Conclusions

A combination of collagen peptide type-1, low molecular weight chondroitin sulphate, sodium hyaluronate, and vitamin-C effectively treats Achilles tendinopathy. The nutraceutical group has a better outcome than the diclofenac sodium group in treating Achilles tendinopathy to reduce pain. The treatment effect on *TA* thickness on USG was not statistically significant between the two groups. However, the percentage reduction of anteroposterior thickness as measured by USG in both transverse and longitudinal probe placement was more in the nutraceutical group.
